# Chiroptera Personality—A Guide for Assessing Personality Variation in Bats: Ecological, Methodological and Statistical Considerations

**DOI:** 10.1002/ece3.74108

**Published:** 2026-07-31

**Authors:** Theresa Schabacker, Mirjam Knörnschild

**Affiliations:** ^1^ Museum Für Naturkunde Leibniz‐Institute for Evolution and Biodiversity Science Berlin Germany; ^2^ Department of Biology, Chemistry and Pharmacy, Institute of Biology Freie Universität Berlin Germany; ^3^ Department of Biology, Faculty of Life Sciences Humboldt Universitӓt zu Berlin Berlin Germany; ^4^ Smithsonian Tropical Research Institute Panama City, Republic of Panama USA

**Keywords:** animal personality, bats, chiroptera, methodological framework, statistical protocol

## Abstract

Consistent between‐individual differences, that is, animal personality (AP) is among the fastest‐growing topics in behavioural biology, with theoretical and empirical work showing important consequences for ecology, evolution and conservation. Yet bats—the second‐largest mammalian order—remain markedly underrepresented despite their functional importance in many ecosystems. We therefore synthesise ecological, methodological and statistical considerations for analysing bat personality while retaining general validity to facilitate transferability to other taxa. Because many bat species exhibit extraordinary life histories and characteristics, we provide structured guidance to design impactful experimental setups, anticipate field‐work challenges, unify schemes for annotating recordings and navigate complex analyses associated with repeated measures. These decisions are critical because they determine repeatability, ecological validity and cross‐study comparability, and they delimit which statistical models can be robustly fitted under realistic sample sizes and field logistics. We provide a comprehensive statistical protocol both as a schematic overview and as an annotated R script accompanied by dummy data. The workflow covers five main steps expected in a hypothetical personality study: (i) analysis of structural covariates using (G)LMMs, (ii) repeatability analysis to identify consistent behavioural measures, (iii) principal component analysis for dimensionality reduction, (iv) validation of traits via control experiments and (v) modelling links from putative behavioural traits to a focal outcome with ecologically significance. Together, these resources enable researchers to follow, understand and implement the entire procedure and to extrapolate the methodology to their own experiments. By unifying methodological practice for a currently under‐represented taxon, we aim to maximise the validity of findings and highlight promising avenues for future studies that establish strong, hypothesis‐driven testing paradigms for bat personalities. More broadly, we seek to deepen understanding of personality variation and its consequences in bats specifically and to enhance the comparative value of AP research generally.

## Graphical abstract

This paper addresses the underrepresentation of bats in animal personality research despite their ecological importance, offering a structured guide for studying consistent individual behavioural differences in this taxon. We outline key experimental, fieldwork and annotation considerations specific to bats' unique life histories, alongside a five‐step statistical protocol—covering mixed models, repeatability, PCA, trait validation and outcome modelling—implemented in an annotated R script including dummy data. The overarching goal is to standardise methodology for maximising the validity of findings and foster strong, hypothesis‐driven testing paradigms for bat personalities.

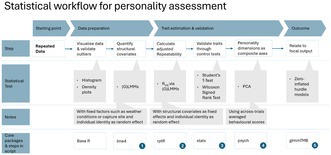

## 1 Introduction

Over the past three decades, research on animal personality (AP), defined as temporally and/or contextually consistent differences in behavioural traits among conspecific individuals, has become a central theme in ethology and behavioural ecology (Carere and Maestripieri 2013). By shifting focus away from a presumed behavioural ‘golden mean’, AP research re‐established individual variation as a key substrate for natural and sexual selection. Early descriptive studies demonstrated that consistent behavioural differences are widespread across taxa (Bell et al. 2009; Réale et al. 2007), enabling hypothesis‐driven research addressing how behavioural variation is maintained and what eco‐evolutionary consequences arise from it (Réale and Montiglio 2021). As a result, AP research now spans ecology, evolution, physiology, conservation and animal welfare.

This rapid expansion initially led to conceptual and methodological fragmentation, with inconsistent terminology and analytical practices hindering comparability across studies (MacKay and Haskell 2015; Sánchez‐Tójar et al. 2022). After extensive debate, the field converged on a consensus definition of personality as consistent between‐individual differences in average behaviour across time and contexts (Dall et al. 2004; Réale et al. 2007). This definition places repeated measurements at the core of personality research and clarifies that personality reflects rank‐order consistency rather than behavioural inflexibility. The concept of behavioural syndromes refers to the consistent covariation among personality traits, either within the same or across different contexts (Dingemanse and Wright 2020). Thus, behavioural syndromes are not synonymous with personality variation but rather emerging from correlations between two or more personality traits. This concept of cross‐contextual repeatability, that is, correlated constraints on behavioural plasticity, is an important notion, as it suggests that for a comprehensive understanding of personality variation in a specific trait (e.g., mating behaviour) it might be necessary to investigate its correlation with other traits within a given context (e.g., mating behaviour with different partners) or across contexts (e.g., contest behaviour or parental care) (Sih et al. 2004).

Most personality studies focus on five broad behavioural traits—activity, exploration, boldness, sociability and aggressiveness—partly inspired by the ‘Big Five’ framework in human psychology (Réale et al. 2007). However, methodological heterogeneity and inconsistent trait labelling have resulted in the jingle–jangle fallacy, where different traits are conflated under the same label or identical traits are split across labels (Carter et al. 2013; Gosling 2001). In response, several influential frameworks have established conceptual clarity and theoretical grounding, enabling major advances in understanding how personality shapes ecological interactions, disease dynamics, movement, survival and reproduction and responses to environmental change (Carter et al. 2013; Dall and Griffith 2014; Dingemanse and Wright 2020; Laskowski et al. 2022; Mackinlay and Shaw 2023; Roche et al. 2016).

Despite this progress, bats (Chiroptera), the second most species‐rich mammalian order, comprising 1500 species, remain strikingly understudied in AP research (Simmons and Ciranello 2025). This gap is particularly concerning given bats' global distribution and their critical ecological roles in pollination, seed dispersal and arthropod control (Kunz and Fenton 2005), as well as the ecosystem services that these functions provide. To date, only 15 studies have examined personality in bats (Table 1), with around half of them focusing on a single family (Vespertilionidae). Currently applied tests involve standard behavioural paradigms such as the hole‐board test (Menzies et al. 2013; Wang et al. 2020), Y‐maze test (Webber and Willis 2020b) and four‐arm test (Boyer et al. 2020), largely borrowed from rodent research. Other bat personality studies used flight tents, for example in open field or novel environment tests (Sagot et al. 2018; Schabacker et al. 2025) or adapted custom‐made devices such as maze arenas or foraging boxes (Harten et al. 2021; Schabacker et al. 2021), likely reflecting attempts to develop more species‐appropriate setups. A few studies assessed bat personalities outside the ‘Big five’ criteria or in (semi‐) natural condition (Nachev and Winter 2019; Sagot et al. 2018). Importantly, research in bent‐wing bats showed that repeatability levels vary significantly with the suitability of the employed test devices and thus, setups such as the hole‐board test originally developed for other (rodent) species, may constrain the correct assessment of personality variation in bats (Kuo et al. 2023). The underrepresentation of bats likely reflects these methodological challenges associated with nocturnality, flight and specialised sensory systems such as echolocation and ultrasonic social communication. However, while taxonomic coverage remains limited, the consistent evidence for personality variation across species and traits suggests it may be widespread among bats.

**TABLE 1:** Studies investigating consistent between‐individual differences in bats.

Authors	Species	Test	Putative traits	Link to
Menzies et al. (2013)	*Myotis lucifugus*	Hole board	Activity, exploration, anxiety	Ontogeny
Chaverri and Gillam (2015)	*Thyroptera tricolor*	In situ/flight cage	Contact calls	—
Sagot et al. (2018)	*Thyroptera tricolor*	In situ/flight cage	Contact calls	Group cohesion
Nachev and Winter (2019)	*Glossophaga commissarisi*	In situ/artificial flower array	Foraging performance	Learning
Boyer et al. (2020)	*Eptesicus fuscus*	Dyadic interactions, four‐arm maze, hole board, open field	Exploration, activity and aggression	—
Wang et al. (2020)	*Vespertilio sinensis*	Hole board	Exploration, activity and aggression	Body mass
Webber and Willis (2020a)	*Myotis lucifugus*	Hole board, Y‐maze test	Exploration, activity, sociability	Roosting behaviour
Webber and Willis (2020b)	*Myotis lucifugus*	Hole board, Y‐maze test	Exploration, sociability	Pathogen transmission
Harten et al. (2021)	*Rousettus aegyptiacus*	Foraging box	Exploration, risk‐taking	Habitat type, learning
Schabacker et al. (2021)	*Pipistrellus nathusii*	Maze arena	Acoustic exploration	—
Kuo et al. (2023)	*Miniopterus fuliginosus*	Hole board, tunnel box, flight tent	Exploration, activity, boldness	—
Kuo et al. (2024)	*Miniopterus fuliginosus*	Hole board, tunnel box, flight tent	Proactivity	Intrinsic and extrinsic states
Fjelldal and van der Kooij (2024)	*Eptesicus nilsonii*	In situ	Arrival date at breeding colony, parturition date	—
Miguel et al. (2024)	*Carollia perspicillata & Artibeus lituratus*	Open field, handling bag, foraging under human presence	Activity, boldness, aggressiveness	—
Schabacker et al. (2025)	*Glossophaga soricina*	Open field, novel object, foraging under risk	Proactivity, exploration, boldness, agitation	Social calling

*Note:* The putative traits we list here are based on the behavioural traits that were originally identified in the respective publications. For reasons of conciseness, we refer to these trait labels, but we strongly recommend that future research rely on operational definitions and avoid assigning predefined labels until the underlying traits have been thoroughly validated.

Beyond methodology, bats' exceptional ecological diversity regarding habitat, prey choice or social systems further complicates the direct application of standard personality paradigms. For example, roosting and foraging preferences critically impact locomotion mode: for some species flight represents the primary and almost exclusive locomotion mode (i.e., the genera *Glossophaga, Choeronycteris* and *Macrotus*) – as opposed to numerous tree‐dwelling species which are used to crawling and exploring small crevices on foot, or even the exceptional quadrupedal gait of species like vampire bats ( *Desmodus rotundus* ), New Zealand's lesser short‐tailed bat *Mystacina tuberculata* and potentially, the hairless bats (*Cheiromeles*) (Schutt and Simmons 2006). Similarly, bats exploit a huge variety of trophic levels, ranging from frugivory, nectarivory and folivory to insectivory, carnivory, sanguivory and omnivory (Barclay and Harder 2003). Such stark differences in foraging preferences impact not only hunting strategies but extend to sensory specialisations and underscore the importance of adjusting behavioural assessments to species' adaptations. Together, these challenges highlight the need for a standardised, yet flexible personality assessment framework to elucidate its ecological and evolutionary relevance.

Here, we synthesise ecological, methodological and statistical best practices into a structured framework for designing personality studies in bats, while maintaining general applicability across taxa. Our aims are to guide assay selection, experimental design and reporting, maximise validity and interpretability, and encourage innovation beyond the traditional ‘big five’ trait repertoire (Dall et al. 2004; Réale and Montiglio 2021). Finally, we provide statistical guidance and an accompanying R‐based protocol with functions, packages and simulated data for quantifying bat personality using repeated‐measures designs (R Core Team 2023), openly available on the Open Science Framework (OSF; https://osf.io/thdq7/overview?view_only=bfd6f9560d6f4f98ac452e115c8b838f) with the goal of fostering rigorous, theory‐driven and comparable research within and beyond Chiroptera.

## 2 Methods

The literature reviewed in this guide was selected non‐systematically, drawing on established key references in animal personality research, behavioural ecology and bat biology, supplemented by targeted searches for specific methodological and statistical topics covered.

### 2.1 General Considerations

**FIGURE 1:**
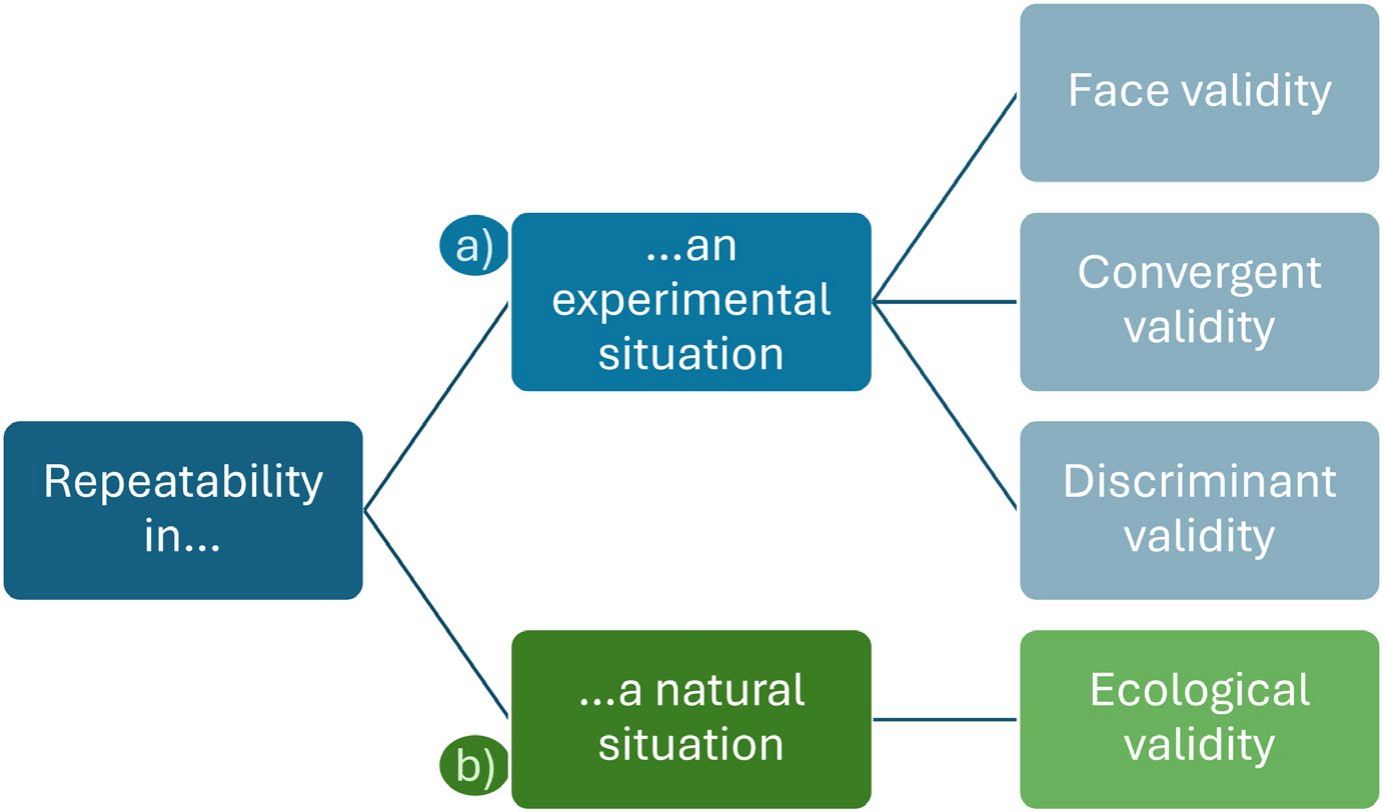
Schematic viewing of general considerations regarding best practices to adhere to in animal personality research: Repeatability should be assessed in (a) an experimental situation, where controlled conditions allow assessment of face, convergent and discriminant validity (the light blue box and following sub‐branches) and (b) in a natural situation to explore ecological validity (the green box and sub‐branch). Here, natural situation refers to conditions reflecting the animal's natural environment without experimental manipulation. Ideally, comprehensive personality assessments quantify individual variation across all levels as they provide complementary forms of validity which cannot be substituted for one another.

Because personality assays are highly context‐ and species‐specific, tests cannot be assumed transferable across taxa or even populations of the same species (White et al. 2020). We therefore outline three core methodological principles that should guide experimental design (Figure 1). First, as personality describes ‘*consistent* between‐individual differences’, repeatability is the defining criterion that must be satisfied for a trait to constitute personality. Across taxa, behavioural repeatability averages around 0.4 (Bell et al. 2009), implying that a single observation predominantly reflects within‐individual variation (~60%), whereas the chances of observing a behaviour reflecting the repeatable component that defines personality is only ~40% (Dingemanse and Dochtermann 2013; Sih et al. 2004). Consequently, personality cannot be inferred from single measurements; repeated exposure is required to estimate consistent between‐individual differences in average behaviour (Dingemanse and Wright 2020). Second, validity must be evaluated at multiple levels, ideally using a multitrait–multimethod framework (Carter et al. 2013). Face validity refers to the plausibility that a test measures the intended behavioural trait based on its design and biological rationale (Gaber 2010). Convergent validity is supported when independent assays that are intended to measure the same trait correlate, whereas discriminant validity is supported when assays targeting different traits do not correlate (Burns 2008; Carter et al. 2013). A major obstacle with repeated measures design are habituation effects that can effectively dilute correct assessments of stable personality traits. Such habituation effects occur especially, though not exclusively, towards novel cues, as with repeated exposure novelty necessarily diminishes. Such effects can be counteracted for example by using different novelty stimuli, for example, presenting a new ‘novel object’ in every trial. Alternatively, changing specific details in the experimental setup can be employed to avoid habituation towards the experimental situation. For example, if boldness is measured as the latency to leave a refuge, the refuge could be constructed by different materials or placed in different spaces between trials. Further, in some cases, for example, assays investigating neophilia or predator responses, appropriate control tests are necessary to evaluate whether the experimental stimuli elicit the expected behavioural responses and that inferred traits are not artefacts of underlying traits or stress. For example, in novelty associated test scenarios, a control test could involve the presentation of a familiar object, achieving familiarity through previous presentation, that is, habituation of the focal individuals to this specific object (see Schabacker et al. 2025; Šlipogor et al. 2016). In other cases, control tests can be an integrative part of the assay itself, that is, quantifying risk‐taking as the difference in emergence times with and without a simulated predator present. Third, ecological validity is particularly critical in bats. Many standard personality assays were developed for laboratory rodents (Hall 1934) and may not yield biologically meaningful results in taxa with distinct sensory systems, locomotion modes and ecological niches. Additionally, the transferability of personality traits between laboratory and field conditions is not fully understood and studies investigation such correlations revealed ambiguous results (Fisher et al. 2015). Contemporary personality research therefore requires that behavioural variation be interpreted in light of species‐specific ecology and, where possible, linked to functional outcomes or behaviour expressed in natural contexts (Carter et al. 2013; Dall and Griffith 2014). Together, these principles ensure that personality assays quantify repeatable, biologically relevant behavioural variation rather than context‐dependent or artefactual responses.

A hypothetical experimental setup respecting the aforementioned considerations could be constructed as follows: we experimentally measure the emergence latency from a safe refuge for a bat species as boldness (face validity), show it correlates with the latency to resume feeding after a predator cue was displayed (convergent validity), but is unrelated to the individual's general level of activity (discriminant validity) and predicts the time of day of emergence from a roost in the wild (ecological validity). A control test should show that the predator cue induces some kind of anti‐predator behaviour in the bats (e.g., freezing, avoidance, aggression), indicating that the stimulus is perceived as risky and thus verifying that what we measure is boldness.

### 2.2 Designing Experiments

#### 2.2.1 Experimental Structure

Recently, biologging and telemetry have been proposed as alternatives to experimental assays to quantify behavioural variation from ‘natural’ behaviours (Hertel et al. 2020). While appealing, such approaches remain constrained in bats: transmitter loads impose substantial flight, thermoregulatory and water costs, rendering many tags unsuitable for small volant species (O'Mara et al. 2014; Speakman and Thomas 2003). Moreover, data from uncontrolled settings are often incomplete and biased due to non‐random sampling, unknown internal states (e.g., hunger, reproductive status, health, social context) and environmental noise that modulates behaviour or limits data quality (Webster and Rutz 2020). We therefore consider standardised behavioural assays essential to minimise extrinsic sources of variation and avoid biased estimates of between‐individual differences (Réale and Montiglio 2021). Following a two‐step approach (Niemelä and Dingemanse 2014), we recommend first quantifying behaviour in controlled, ideally semi‐natural assays that mimic key ecological features while limiting external noise, and second validating assay‐based personality differences by linking them to behaviour expressed in the wild within the same individuals (e.g., Herborn et al. 2010). Crucially, experimental assays should be designed around pre‐defined ecological trade‐offs expected to shape personality variation, ensuring conceptual consistency between experimental and field contexts (Dall and Griffith 2014).

#### 2.2.2 Experimental Design

Behavioural assays must be tailored to species‐specific adaptations that may compromise the validity of standard tests. Bats differ fundamentally from commonly studied model taxa and show substantial interspecific heterogeneity in locomotion, ecology and sensory specialisation. For example, vertical test designs may bias results due to bats' general tendency to move upwards, while small arenas that restrict flight can be inappropriate for species for which flight is the primary locomotor mode (Menzies et al. 2013; Wang et al. 2020). Accordingly, experimental setups should reflect species‐specific locomotion, wing morphology and movement ecology when determining arena size and structure (Wang et al. 2020). Habitat use and hunting strategy further shape perceived risk: tests involving exposure or openness may elicit qualitatively different responses in open‐air foragers compared to clutter‐adapted species (Lima and O'Keefe 2013). Consequently, neither methods nor results from species‐specific assays are directly transferrable across species. However, this limitation is an inherent consequence of prioritising ecological validity: species‐specific insight can only be gained through species‐specific methodology, and researchers should therefore take advantage of the established knowledge regarding bat ecology (see for example Altringham 2011; Dietz and Kiefer 2018; T. Kunz and Fenton 2005; Neuweiler 2000). Test stimuli and assay design should therefore be unambiguous and ecologically relevant. Before assay design, we recommend explicit consideration of feeding ecology, movement patterns, social structure and specialised morphological adaptations. Additionally, sex, body condition, reproductive or nutritional status can significantly influence personality traits and their modulation by other factors (Bouchebti et al. 2022; Kuo et al. 2024). Consider, for example, conducting sex‐specific assays, standardise nutritional conditions as much as possible and meticulously measure and statistically control for variations in these factors (see below). Finally, bats' reliance on echolocation and social vocalisations offers additional behavioural dimensions: consistent inter‐individual differences in vocalisation rates have been documented in several species and may represent a promising personality axis (Sagot et al. 2018; Schabacker et al. 2021, 2025).

A crucial experimental detail relates to the length of the inter‐trial interval (ITI), that is, the time interval between repeated measures: longer ITIs generally increase reliability but often decrease repeatability, partly because they reduce temporal autocorrelation (Bell et al. 2009). Temporal autocorrelation refers to the increased likelihood of behavioural measures being more similar if they are taken closer together in time. It can arise, for example, through intrinsic (hormone levels, metabolic rates) or extrinsic (environmental factors, seasons) states and has the potential to confound personality estimates by inflating repeatability values, hence it is also termed ‘pseudo‐replication’. While level and risk of autocorrelation heavily depend on the system, research questions and project constraints, a general recommendation is to collect enough data to statistically account for autocorrelation by incorporating a temporal autocorrelation parameter in the models (but see Mitchell et al. 2020). From an experimental design perspective, temporal autocorrelation is best prevented by using the longest ITIs possible. However, personality is expected to change across very long time scales or across critical developmental stages (Cabrera et al. 2021). Additionally, for wild animals in captivity, long ITIs can be unethical and limited by field related constraints. Thus, a highly species‐ and context‐specific trade‐off must be struck between welfare and data quality.

### 2.3 Conducting Experiments

#### 2.3.1 Fieldwork Challenges

As personality traits such as boldness influence capture probability (Biro and Dingemanse 2009; Wilson et al. 1993), sampling bias is likely to occur. We recommend combining capture methods (e.g., roost capture and mist‐netting) and adhering to the STRANGE framework to mitigate bias (Webster and Rutz 2020). Bats' fission–fusion dynamics complicate recapture and repeated testing, making short‐term captivity a practical solution in many studies. However, species selection is critical, as not all bats tolerate temporary captivity equally; husbandry feasibility should therefore be assessed in advance. Bats should be housed in appropriate flight cages with respect to species' body size and flight dynamics. Flight cages should be placed in undisturbed, shaded areas and always contain artificial roosts equipped with material to cling to. For individual identification it is necessary to mark bats at least temporarily and should always be done with the least invasive method feasible. Established methods are for example wing punches, which typically heal between 16 and 120 days depending on species and biopsy punch size, and are not known to affect flying ability or alter behaviour (Loeb et al. 2026). Passive‐Integrated‐Transponders (PIT‐tags) are subcutaneously injected tags with a unique number that allow permanent identification, if applied correctly. Irritations, infections and hair loss can occur at the injection site, so wounds should be monitored carefully after application. Bands, collars and radio transmitters carry a greater risk of (sub‐)lethal effects on bat health and behaviour and should, if used at all, only be applied by trained and experienced personnel (Loeb et al. 2026). After capture and marking, individuals should be given habituation time while closely monitoring welfare indicators. Indicators should span physiological measures—such as body weight or hydration status via skinfold thickness (Kavouras 2002; Pearce et al. 2008) – and behavioural measures, including feeding patterns, stereotypic or non‐functional behaviours and inappropriate time‐budgeting like excessive grooming (Wolfensohn et al. 2018). Social calling should also be monitored, as high vocalisation frequencies have been associated with elevated cortisol levels in some bat species (Edwards et al. 2022; Freeman et al. 2018). Experimental designs should counterbalance test order, include rest periods and track body mass regularly (Bell 2013). Feeding status should be standardised without extra fasting to avoid stress‐induced behavioural artefacts, particularly in species with high energetic turnover (Amitai et al. 2010; Bechler et al. 2024). Testing should minimise prior stressors and, where possible, be conducted in semi‐controlled environments to reduce environmental noise and influences that might modulate individual behaviour (Perks and Goodenough 2020). Test arenas should also be shielded from external conspecifics, whose vocalisations may disrupt behavioural assays.

#### 2.3.2 Recording and Annotating

Conducting behavioural assessments in controlled environments, that is, test arenas, offers the advantage of video recording the experiments and observed behaviour. This method enables cross‐validation among observers, allows detection of brief or subtle behaviours, and ensures reliable behavioural analysis, especially if more than one behavioural measure is extracted within the same experimental setup. Direct observation alone is often insufficient due to observer bias and limited temporal resolution. Camera setups should match expected behaviour types; in larger arenas this may require combining overview recordings of locomotion with close‐up views of focal zones. Behavioural annotation can be performed using software such as BORIS (Friard and Gamba 2016), which supports flexible ethograms and event‐ or state‐based scoring. Automated tracking programs like DeepLabCut (DLC) and related tools use deep neural networks to precisely track animal movements by user‐defined features with impressive accuracy (Nath et al. 2019), effectively removing observer bias and enabling high‐throughput, fine‐grained behavioural scoring. However, due to its high complexity and dynamism flight has been notoriously difficult to track automatically, with bat flight posing an especially challenging task, even for AI based tools (Håkansson et al. 2024). Furthermore, training robust models requires large, annotated datasets that do not yet exist for most bat species. Nevertheless, very recently Håkansson et al. (2024) developed a 3D videography workflow that successfully tracked bats in both a wind tunnel and a large free‐flight enclosure. Such technical advances promise to reduce the labour‐intensive manually digitisation of tracking and scoring bat behaviour in video recordings and should be considered in future research, albeit with non‐trivial technical and computational demands.

### 2.4 Analysing Data

Prior to analysing recordings, ethograms and behavioural variables should be defined with the help of preliminary observations to understand species‐specific expression. During these preliminary observations, attention should be drawn to behaviours that are both reliably observable and feasible to score within time constraints. Behavioural variables should be chosen with careful consideration of the ecology and natural behaviour of the bat species in question, requiring the observer to operate with flexibility and biological insight. Behavioural units should be scorable independently of prior behaviour, that is, avoid variables that rely on stimulus exposure only after a certain condition is met (e.g., capturing behavioural response towards a playback which starts after aggressive behaviour was expressed).

Various types of variables can be assessed: Count variables, that is, events in a window (e.g., number of interactions with a novel object); Rates, that is, counts per time (e.g., number of zone entries per minute); Binary variables, that is, success or failure of a behaviour (e.g., approached vs. not); Latency measures, that is, duration until a behaviour is scored (e.g., time until emergence from a shelter), Proportions, that is, success of trials (e.g., time spent in open area out of total time); Ordinal scores, that is, counts on a scale (e.g., 0–5 of aggressiveness). Each set of variables is accompanied by specific statistical considerations which must be accounted for in the statistical analyses (see below).

### 2.5 Repeatability & Statistical Analysis

Statistically, repeatability is considered the fraction of the total phenotypic variance that can be attributed to variation among individuals, assessed through the common measure of *R*:

(1) $$R=\frac{V_{A}^{2}}{V_{A}^{2}+V_{W}^{2}}$$

where $V_{A}^{2}$ accounts for the variance among individuals, and $V_{W}^{2}$ represents the within‐individual variance. The sum of both variance terms thus represents the total phenotypic variation $V_{P}^{2}=V_{A}^{2}+\;V_{W}^{2}$ present in a given population (Nakagawa and Schielzeth 2010; Réale and Montiglio 2021). Therefore, how repeatable a trait is, depends on the variance present in the population, that is, individual repeatability will only be evident, if there is measurable variation between individuals that is substantial relative to within‐individual variation. In other words, repeatability will be high, when within‐individual variation is low compared to between‐individual variation. Thus, it is useful to first compute descriptive statistics to understand the level of $V_{P}^{2}$ present in the data set. Data outliers should be re‐evaluated for validity. However, if they do not arise from obvious measurement error, they should be retained, as they capture the very variation personality research aims to explain. Following, *R* can be assessed through correlations, one‐way ANOVA and linear mixed‐effects models, which are currently regarded as the gold‐standard, as they allow the partitioning of variance in $V_{A}^{2}$ and $V_{W}^{2}$ (Dingemanse and Dochtermann 2013; Laskowski et al. 2022; Nakagawa and Schielzeth 2010). Nakagawa and Schielzeth (2010) describe several types of repeatabilities: (1) agreement repeatability refers to the reproducibility of measures in absolute terms, while (2) adjusted repeatability accounts for confounding factors by including fixed effects (e.g., environmental, intrinsic or treatment effects). Factors such as sex, body mass or reproductive state can alter behavioural expression and can be accounted for if integrated as fixed effects. Thus, including structural confounding factors will change the variance components and hence the repeatability, and is the repeatability estimate most often used in personality studies. (3) Conditional repeatability calculates repeatability depending on a certain value of a fixed factor, for example, *R* at a specific age. The *rpt:R* package (Stoffel et al. 2017) facilitates the computation of either repeatabilities and provides guidance when dealing with non‐Gaussian data, in which case GLMMs present a powerful tool. In any case, several assumptions associated with (G)LMMs represent crucial pitfalls and while we point out a number of considerations below, analyses should build upon careful reading of core references (e.g., Bolker et al. 2009; Dingemanse and Dochtermann 2013; Nakagawa et al. 2017; Nakagawa and Schielzeth 2010; O'Dea et al. 2022; Schielzeth et al. 2020): Valid inferences require an appropriate family–link specification; misspecification can bias both *R* and variance components and should be chosen based on theoretical justification and addressed via diagnostics. In addition, models assume linearity on the data scale (LMM) or link scale (GLMM), absence of problematic multicollinearity and conditional independence of residuals. LMMs also assume homoscedastic residual variance, whereas GLMMs assume the variance follows the distribution's mean–variance relationship, so over/under‐dispersion and zero inflation must be checked and modelled when present. This occurs predominantly with count and rate variables, which often contain excess zeros. While count variables can be sensitive to a few very active individuals, rate variables can suffer from truncation. Binary variables can contain low information per trial and risk of low variation between individuals. Latency variables can be skewed because many animals never respond within the time limit (but would do so later) and should thus be modelled as survival curves, which can be less intuitive to report. Proportion data can be misleading because animals often have different chances to respond and can lead to many trials with 0% or 100%, so models must be chosen with the right likelihood to account for overdispersion and boundaries. Ordinal scores can be highly subjective to score and suffer from rater effects and can convert small underlying differences into disproportionate category shifts. These conditions require an informed model choice and running model diagnostics is imperative—including checks for residual normality, homogeneity of variance, linearity and outliers—and we provide a reproducible protocol for these steps in the ESM. Even though mixed models are surprisingly robust towards some violations (e.g., non‐normality of fixed effects), robustness is context‐dependent rather than universal as heteroscedasticity and dependence can distort standard errors and variance components (Schielzeth et al. 2020). Thus, correctly reporting the chosen measure of *R* along with associated uncertainty, that is, confidence intervals (CI), both variance terms and the mixed model's fixed and random effects (estimates and CI) are key for any personality study (Dingemanse and Wright 2020; Nakagawa and Schielzeth 2013). Additionally, report whether *R* is estimated on the latent (link) or observation scale, as this affects interpretation.

Choosing an appropriate sample size and sampling regime presents a continuous challenge in personality research and poses an important question as the chances to statistically estimate *R* correctly, largely depends on the ‘true’ repeatability of a given trait in the population and the number of individuals sampled (Réale and Montiglio 2021). However, resulting from time‐constraints, researchers often face a three‐dimensional trade‐off between number of individuals (*n*) vs. number of repetitions (*k*) vs. number of experiments. Higher *k* will reduce the measurement error around *R*, however, it is unlikely to greatly change the estimate itself (Bell et al. 2009). A greater number of experiments allows estimation of cross‐contextual validity and convergent validity. While a higher *n* is always admirable, as it allows for enhanced statistical analysis such as multivariate hierarchical testing (see below, Dingemanse and Dochtermann 2013), logistical constraints in the field often do not permit such high numbers of experimental subjects. We thus recommend piloting one assay to estimate covariances, if pilot shows moderate R (> 0.4), aim for *k* = 2–3 and *n* with a minimum of 40, better 60–100 individuals (Martin et al. 2011; Van De Pol 2012), but see Wolak et al. (2012). Preliminary power analysis can aid in sample size determination (e.g., Arifin 2018), as well as the R package *ICC*, which is specifically tailored towards estimating *n* for a given repeatability value with a desired confidence interval width (Wolak et al. 2012). Depending on the research question, 2–3 assays per putative trait can be employed to confirm convergent or discriminant validity or investigate nuances between seemingly similar behaviours (e.g., behaviour during novel object exposure vs. novel food exposure, both presumably capturing individual neophilia/neophobia levels). However, if the aim is to investigate behavioural syndromes, links to life history, ecological traits, environmental plasticity, or conservation applications broad personality‐typing through a battery of different experiments is most useful (Carter et al. 2013; Réale et al. 2007). In this case, we recommend testing fewer assays per trait for a greater number of traits.

Multivariate (co)variance models and double‐hierarchical mixed models offer enhanced resolution by estimating multiple traits jointly (e.g., behavioural‐syndrome structure, reaction norms) and by modelling both means and dispersion (Dingemanse and Dochtermann 2013; Mitchell and Houslay 2021; O'Dea et al. 2022). When fitted in a Bayesian framework, they provide full posterior uncertainty for the derived estimates/quantities (e.g., *R*, individual (co)variances) (Nakagawa and Schielzeth 2010; O'Dea et al. 2022). Both frequentist and Bayesian implementations can also accommodate overdispersion and zero inflation, problems which are common in personality datasets (Brooks et al. 2017; Zuur et al. 2009). Unfortunately, these models are extremely data hungry, so achieving adequate *n* and *k* is often challenging in ecological field studies (Garamszegi and Herczeg 2012), especially in wild bats. We therefore encourage a simulation‐based study design, to assess feasibility, required sample sizes and expected precision before data collection (for guidance see Johnson et al. 2015; Kain et al. 2015; Kelter 2024; Van De Pol 2012).

Principle Component Analysis (PCA) condenses large data into a small set of features with minimal loss of information (Kherif and Latypova 2020). This dimension‐reduction method constructs new Principal Components (PCs) which represent the original data and maintain most of its variation. These PCs can be used in further analyses and thus reduce problems like multicollinearity and the number of statistical tests needed when testing hypothesis. Original variables that load onto the same PCs are assumed to share underlying behavioural mechanisms (Budaev 2010). Thus, PCA is a useful and widely used tool in personality research to extract information about individual differences, for example when multiple traits within one experiment have been assessed or to understand shared traits across multiple experiments, that is, a behavioural syndrome structure. Importantly, PCA should never be used on repeated measures (Budaev 2010) and valid critique and advisory towards this method have been brought forward, see for example Dingemanse et al. (2010) and Del Giudice (2020, ESM S1). Limitations include for example sensitivity to small sample sizes and researchers' decisions about scaling, rotation, number of components retained and labelling, which can alter the results obtained and thus introduce a subjective bias. Nevertheless, PCA represents a powerful tool to reduce dimensionality when applied carefully, for example through cross‐method comparison or robustness checks (Ramos et al. 2022). Thus, careful reading of required pre‐ and post‐diagnostics is imperative (see for example Budaev 2010).

### 2.6 Hypothetical Experiment and Statistical Protocol

**FIGURE 2:**
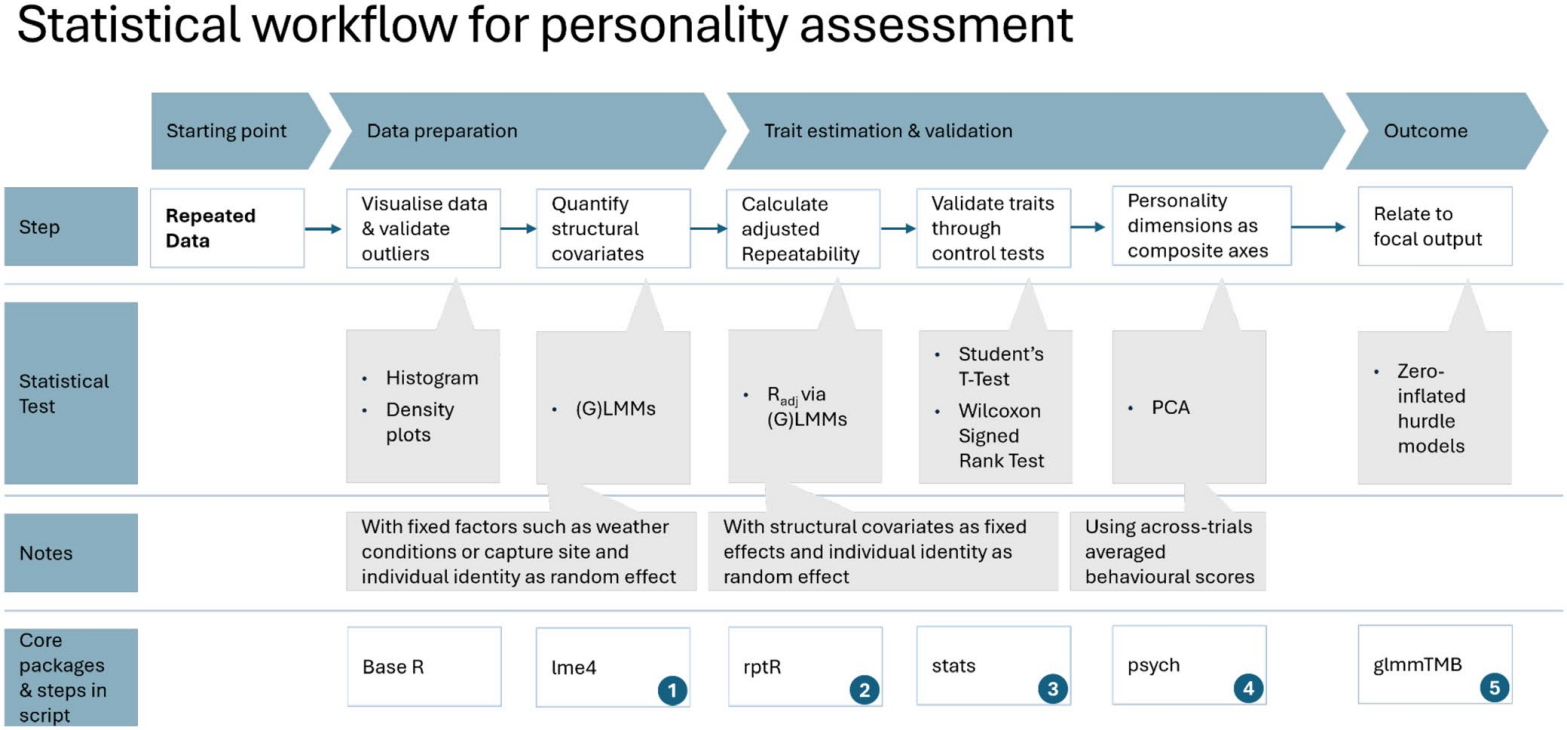
Statistical workflow visualising the suggested procedure for analysing personality variation. The swim lane diagram demonstrates the different steps from the analysis, which statistical tests to use, notes on specific details and which R packages to use. The numbers in blue circles indicate the section number in the accompanying R script (see ESM S2).

To illustrate the proposed workflow (Figure 2), we applied the full statistical protocol to a simulated dataset representing a hypothetical bat personality study. The dataset consists of 40 individuals, each subjected to four semi‐natural behavioural assays conducted in a flight tent—latency to emerge from a refuge, latency to resume feeding after a predator cue, a predator cue control test and general activity in a known environment—as well as one test conducted under natural conditions, namely emergence from a roost in the wild. Each individual completed three repeated trials per test, with trials separated by two‐week intervals, resulting in a repeated‐measures structure suitable for repeatability estimation. The workflow proceeds in five steps. First, we fit linear mixed‐effects models to quantify whether structural covariates (e.g., capture site, individual features, habituation process) impact the behavioural readouts (step 1). Second, we estimate adjusted repeatability (*R*_adj_) for each behavioural readout across trials and contexts using mixed‐effects models with significant fixed effects retained, reporting point estimates and confidence intervals (step 2). *R*_adj_ is computed using the rptR package (Stoffel et al. 2017), which partitions phenotypic variance into among‐individual and within‐individual components and calculates R as shown in Equation (1) after accounting for fixed effects; confidence intervals are obtained via parametric bootstrapping and *p*‐values via permutation of individual identity across observations. Third, we validate behavioural assays by comparing treatment and control conditions using paired tests (step 3). Fourth, we conduct principal component analysis on averaged variables to derive composite behavioural axes, aiding interpretation of putative personality dimensions (step 4). Fifth, to relate behavioural tendencies to a focal output characterised by excess zeros and positive counts, we apply zero‐inflated hurdle models to model occurrence and frequency components separately (step 5). Across all steps, routine diagnostics are performed including inspection of residual structure, overdispersion and multicollinearity. For a detailed description of the hypothetical experiment please see the Supporting Information S1 (ESM S1, S3). All analytical steps above are implemented in a fully annotated R protocol providing necessary packages and functions, and demonstrating the applicability of the protocol using our dummy data from the hypothetical experiment, available on the Open Science Framework (OSF; https://osf.io/thdq7/overview?view_only=bfd6f9560d6f4f98ac452e115c8b838f).

## 3 Results

### 3.1 Reporting Personality Variation

Repeatability estimates are sensitive to several factors, such as *n*, *k* and ITI. Without a thorough reporting of these details, the reliability of these estimates diminishes. Additionally, when reporting repeatability estimates, it is crucial to present the type of repeatability, for example, adjusted R, alongside it's estimate, credibility interval and *p*‐value, among (*V*_*ind*_) and within (*V*_*e*_) individual variance components (Dingemanse and Wright 2020). Reporting confidence intervals is of high importance as identical (point estimates) of R can reflect vastly different levels of precision and biological relevance, risking the substitution of one ‘golden mean’ for another. Further, as we mentioned earlier, repeatability is a prerequisite, not a conclusion and should be treated as the starting point for further investigations, requiring explicit and thorough reporting.

Additionally, personality estimates should always be accompanied by all fixed and random effect parameters estimates, alongside their uncertainties, that is, credible intervals and information on the number of groups and repeated measures (*n* and *k*).

### 3.2 Dealing With Behavioural Data

As outlined above, there is an inherent importance of validating the meaning of behavioural traits, if we want to link them correctly to ecological function or evolutionary theory (Carter et al. 2013). Importantly, behaviour is shaped by internal state, environmental conditions and test structure, all of which must be accounted for before attributing observed patterns to consistent personality. Moreover, behaviour should not be automatically labelled as representing a specific trait (e.g., ‘boldness’ or ‘activity’) without examining the ecological context and the structure of the test. Instead, operational definitions should be preferred: rather than labelling a behaviour as ‘boldness’, describe it as ‘latency to emerge from a shelter’ (Laskowski et al. 2022). Additionally, the same behaviour can represent different traits in different contexts, consider for example important differences in behavioural responses when using voluntary vs. forced exposure (Carter et al. 2013). Novel environment assays are often labelled as activity/exploration tests, however, when the individual is forced to enter the novel arena, these tests are more likely to index stress‐coping style rather than general activity or exploration (Koolhaas et al. 1999). Thus, high levels of locomotion in a forced novel environment assay might be indicative of an individual's stress level, whereas similar locomotion in a familiar or voluntarily entered environment might be more plausibly representing exploration levels (Burns 2008; Finger et al. 2016). Accordingly, assay design (e.g., voluntary entry, acclimation time or familiarity) should be reported explicitly and validated via convergent measures before assigning trait labels. Importantly, behavioural variables must be interpreted through an ecological lens, not just via generalised frameworks and cross‐context and/or cross‐species generalisation should be avoided. While we recommend predefining expected trait categories to guide test development, the attribution of specific behaviours to traits should be part of the discussion (Laskowski et al. 2022). Considering which behavioural measures are repeatable, which variables cluster together (e.g., in PCA) and whether they form a consistent trait structure will prevent circular reasoning and over‐interpretation of raw behaviour.

### 3.3 Hypothetical Experiment

Please note: all results presented in this manuscript derive from simulated dummy data generated for illustrative purposes and are provided alongside the R protocol on OSF (ESM S1, S2, S3). No empirical data were collected for this study. The results are intended to demonstrate the analytical workflow rather than to draw biological conclusions about bat personality.

In our hypothetical experiment, structural covariates including weight, capture site and repetition did not significantly influence the behavioural readouts (Table 2). Both exemplary variables yielded moderate to high adjusted repeatability estimates with narrow confidence intervals, suggesting reliable individual differences in refuge emergence behaviour (Table 3). Tables 2 and 3 are presented in the main manuscript as exemplary outputs to guide reporting, as they represent important steps for repeatability estimation; full results from all analytical steps are provided in the ESM. Bats showed significantly more anti‐predator behaviour in response to the predator cue than to the conspecific cue, confirming that the predator stimulus was perceived as a genuine risk signal (ESM Figure S1). PCA successfully reduced behavioural variables into interpretable personality axes (ESM Table S1). Correlations between PCs confirmed convergent validity between refuge emergence behaviours and feeding after a predator cue, while the absence of a correlation between refuge emergence behaviour and general activity variables confirmed discriminant validity (ESM Figure S2). Hurdle models further showed that both refuge and feeding PC1 significantly predicted roost emergence latency in the wild (ESM Table S2).

**TABLE 2:** GLMM results from fixed effects (structural factors, ‘Predictors’) and random effects (bat identity) parameter estimates from two exemplary behavioural measures from our hypothetical personality study, calculated from the dummy data available in the ESM.

Predictors	Time until full body out			Number of returns		
	Est.	CI	*p*	IRR	CI	*p*
(Intercept)	4.58	4.04 to 5.12	**< 0.001**	1.72	1.06–2.77	**0.027**
Weight	0.10	−0.15 to 0.34	0.427	1.13	0.91–1.40	0.276
Capture Site [Site_B]	0.13	−0.52 to 0.77	0.701	0.86	0.49–1.51	0.592
Capture Site [Site_C]	0.05	−0.60 to 0.70	0.879	1.01	0.58–1.77	0.973
Repetition [2]	0.20	−0.14 to 0.54	0.253	1.25	0.93–1.69	0.134
Repetition [3]	0.19	−0.15 to 0.53	0.277	1.19	0.88–1.60	0.265
*Random effects*						
*σ* ^2^	0.59			0.37		
*τ*_00_	0.44_Bat_ID_			0.32_Bat_ID_		
ICC	0.43			0.46		
N	40_Bat_ID_			40_Bat_ID_		
Observations	120			120		
Marginal *R* ^2^/conditional *R* ^2^	0.020/0.438			0.042/0.480		

*Note:* Bold values indicate statistically significant estimates (*p* < 0.05).

Abbreviations: CI = confidence interval, Est. = estimate, IRR = incidence rate ratio.

**TABLE 3:** Reporting repeatability estimates.

Test	Variable	*R*_adj_	95% CI lower—upper	*p*	*V*_ind_ ± SD	*V*_e_ ± SD	*N* Obs.	*N* groups	LRT/Perm.
Refuge	Time Until Full Body Out	0.51	0.29–0.66	0.002	0.39 ± 0.62	0.59 ± 0.63	120	40	Perm.
	Number Of Returns	0.47	0.20–0.63	0.002	10.95 ± 3.31	—	120	40	Perm.

*Note:* Results from our hypothetical experiment calculated with the rpt() function from the rptR package (Stoffel et al. 2017) with the dummy data, details provided in the ESM.

Abbreviations: Obs. = observation, Perm. = permutation, R_adj_ = adjusted repeatability, V_e_ = within‐individual variance component, V_ind_ = among‐individual variance component.

## 4 Discussion: Integrating Personality With Ecological and Evolutionary Functions

To demonstrate the applicability of the proposed workflow, we applied it to a simulated dataset exemplifying a bat personality study. The results illustrate how repeatability estimates, variance components, control tests and challenging datasets can be analysed and reported in a standardised way. Importantly, the workflow successfully recovered the simulated individual differences, demonstrating its sensitivity and practical applicability even under the constraints commonly faced in chiropteran (field) research, such as limited sample sizes and skewed data distributions.

Establishing repeatable individual differences is only the first step in animal personality (AP) research. To generate biologically meaningful insights, emergent personality variation must be linked to ecological and evolutionary processes that both shape and are shaped by between‐individual variation. Below, we highlight conceptual frameworks that are particularly promising when applied to personality variation in bats.

The pace‐of‐life syndrome hypothesis predicts covariation among life‐history, behavioural and physiological traits (Réale et al. 2010), while the functional syndrome framework extends this idea by explicitly linking correlated response and effect traits to ecosystem processes (Raffard et al. 2017). Response traits describe how individuals interact with their environment, whereas effect traits describe how individuals modify ecosystem functioning through processes such as resource use, energy flow, or nutrient transfer (Raffard et al. 2017). This framework places individual variation at the core of eco‐evolutionary dynamics. Given bats' major contributions to pollination, seed dispersal and insect suppression (Kunz and Fenton 2005), consistent differences among individuals in space use, dispersal or foraging strategies could have disproportionate effects on forest regeneration, plant diversity, or food‐web structure. While these relationships have been extensively explored in insects (pollination, e.g., Burns 2005), rodents (seed dispersal, e.g., Brehm et al. 2019) or predatory species (pest control, e.g., Royauté et al. 2015), to date, there is no study exploring these relationships in bats, albeit the taxon of bats encompassing all of the above‐mentioned traits. This highlights a clear avenue for future research, with structured approaches now available to quantify such links (Hunter et al. 2022).

A related framework is the concept of keystone individuals, which posits that certain individuals disproportionately influence ecological processes (LaBarge et al. 2024). In bats, individuals with particular behavioural profiles may exert outsized effects on interacting species or ecosystem processes, for example through consistent movement or foraging patterns (LaBarge et al. 2024). Identifying such individuals requires individual‐based approaches, including social and interaction networks (Krause et al. 2010; Snijders et al. 2014; Tonos et al. 2022). Bats are especially well suited for this line of inquiry due to their extraordinary diversity of social systems, which range from solitary lifestyles to large, highly structured colonies with complex social interactions (Kerth 2008).

Although bat personality research is still scarce, existing studies already suggest that individual differences both structure and are structured by social life. For example, consistently more vocal individuals in *Thyroptera tricolor* play a key role in group cohesion and roost discovery (Sagot et al. 2018), while more sociable male *Myotis lucifugus* acquire higher pathogen loads through increased social contact (Webber and Willis 2020b), with likely fitness consequences. These findings underscore the value of integrating personality with social network approaches, for which established conceptual roadmaps are available (Jolles et al. 2020).

Finally, personality variation must be considered in conservation planning to maintain ecosystem function and resilience (Brodie et al. 2018). Bat populations are declining globally, and with the majority of species requiring conservation attention (Frick et al. 2020), targeted and effective management strategies are urgently needed. There is growing evidence that conservation outcomes—such as post‐translocation survival, habitat use, stress responses and reproductive success—are strongly influenced by personality variation (Mackinlay and Shaw 2023; Merrick and Koprowski 2017). This reinforces the importance of studying personality in wild bat populations, particularly in contexts directly relevant to conservation decision‐making.

Together, these frameworks are highly useful for hypothesis‐driven research on bat personality and its ecological consequences. Future work should assess the comparability of personality measures across species, ecological guilds and experimental contexts, and foster collaborative efforts to validate behavioural assays across bat taxa with diverse life histories. While species‐specific assay design limits direct methodological comparability, cross‐taxa analyses can nonetheless help clarify how different selective pressures shape expressed traits and illuminate trait‐ecology relationships with their functional implications (White et al. 2020). Such comparative approaches will deepen our understanding of personality variation in bats while contributing to broader AP research.

## Author Contributions

**Theresa Schabacker:** conceptualization (equal), data curation (lead), formal analysis (lead), funding acquisition (equal), investigation (lead), methodology (lead), project administration (supporting), resources (supporting), validation (equal), visualization (lead), writing – original draft (lead), writing – review and editing (lead). **Mirjam Knörnschild:** conceptualization (equal), data curation (supporting), formal analysis (supporting), funding acquisition (equal), methodology (supporting), project administration (equal), resources (lead), software (lead), supervision (lead), validation (equal), visualization (supporting), writing – review and editing (equal).

## Funding

This work was supported by the Elsa‐Neuman stipend from the state of Berlin, Germany, to T.S. and by grants from the European Research Council under the European Union's Horizon 2020 Programme (ERC GA 804352) and the Leibniz Foundation (P122/2020) to M.K.

## Conflicts of Interest

The authors declare no conflicts of interest.

## Supporting information


**Figure S1:** Step 3—Wilcoxon signed rank test from predator cue test. Plots depicting the mean number of (1) freezing events, (2) total time frozen and (3) number of aggression calls during the conspecific or predator condition, respectively (*n* = 40). The grey horizontal bar in the boxplots depict the mean and the grey vertical bars outside the boxplots depict the 95% confidence intervals. *p*‐values of the Wilcoxon Signed Rank Test are given in each panel for the respective test.
**Figure S2:** Correlation between PCs across tests. Refuge_PC1 is representative for a refuge boldness axis, as it is mainly constructed by variables related to the time leaving the safe refuge. Feeding_PC1 is representative for a feeding boldness axis, as the variables loading onto this component are related to antipredator behaviour and the latency to resume feeding after the simulated predator attack. Panel A shows that refuge boldness and feeding boldness correlate, indicating that we can confirm convergent validity. Panel B shows now correlation of Refuge_PC1 with Activity_PC1 which represents the general activity axis of an individual, as the component is constructed by variables related to general locomotion. The lack of correlation in Panel B thus confirms discriminant validity.
**Table S1:** Results from PCA for the three experimental assays. PCA was varimax rotated.
**Table S2:** Results from hurdle models used to assess the relationship between personality traits (captured as PCs) and latency to emerge from the natural roost. IRR = Incidence Rate Ratios, CI = Confidence Intervalls, *p* = *p*‐value. Significant values are in bold. *N* = 40, *k* = 3.

## Data Availability

All data and code to reproduce the presented analytical steps are implemented in a fully annotated R protocol including the dummy data from the hypothetical experiment, available on the Open Science Framework (OSF; https://osf.io/thdq7/overview?view_only=bfd6f9560d6f4f98ac452e115c8b838f).
