# Supplementary material for: Chiroptera Personality—A Guide for Assessing Personality Variation in Bats: Ecological, Methodological and Statistical Considerations
Source: Ecol Evol. 2026 Jul 31;16(8):e74108. doi: 10.1002/ece3.74108 (PMC13427613; doi:10.1002/ece3.74108)
Supplement: Supplementary file 1 — Figure S1: Step 3—Wilcoxon signed rank test from predator cue test. Plots depicting the mean number of (1) freezing events, (2) total time frozen and (3) number of aggression calls during the conspecific or predator condition, respectively (n = 40). The grey horizontal bar in the boxplots depict the mean and the grey vertical bars outside the boxplots depict the 95% confidence intervals. p‐values of the Wilcoxon Signed Rank Test are given in each panel for the respective test. Figure S2: Correlation between PCs across tests. Refuge_PC1 is representative for a refuge boldness axis, as it is mainly constructed by variables related to the time leaving the safe refuge. Feeding_PC1 is representative for a feeding boldness axis, as the variables loading onto this component are related to antipredator behaviour and the latency to resume feeding after the simulated predator attack. Panel A shows that refuge boldness and feeding boldness correlate, indicating that we can confirm convergent validity. Panel B shows now correlation of Refuge_PC1 with Activity_PC1 which represents the general activity axis of an individual, as the component is constructed by variables related to general locomotion. The lack of correlation in Panel B thus confirms discriminant validity. Table S1: Results from PCA for the three experimental assays. PCA was varimax rotated. Table S2: Results from hurdle models used to assess the relationship between personality traits (captured as PCs) and latency to emerge from the natural roost. IRR = Incidence Rate Ratios, CI = Confidence Intervalls, p = p‐value. Significant values are in bold. N = 40, k = 3. [file ECE3-16-e74108-s001.docx]

Supplementary Information – Chiroptera Personality

**S1. Exemplary experimental set up**

The experimental design of our hypothetical test scenario consists of five behavioural tests. Four tests are conducted as standardised behavioural assays in a semi-natural environment, and one additional test is carried out in the wild. Note that as we focus on the statistical protocol here, the experiment is not set up in all details.

Bats were captured from the wild and kept in temporary captivity. During this period, the four standardised assays were conducted in a large flight tent. A total of 40 bats was tested. Each individual received a unique Bat ID, which is included as a variable in the dataset. For all tests (including the wild test), each bat underwent three trials (repetitions). The three repetitions of a given test were always conducted on different days, with an interval of two weeks between each repetition. All four semi-natural tests have a fixed duration of 15 minutes each and were carried out as follows:

*Latency to Emerge from a Refuge*. In this test, bats are placed in a safe refuge that is familiar to them. The following variables are recorded: “Time Until Head Out” measures the time until the bat first protrudes its head from the refuge. “Time Until Full Body Out” measures the time until the bat exits the refuge completely with its entire body. “Number of Retractions” counts how many times the bat moves back into the refuge and out again, including partial retreats and re-emergences, before leaving the refuge fully. “Number of Returnings” counts how many times the bat moves back inside the refuge after it has left the refuge fully for the first time. “Total Time In Refuge” refers to the cumulative time an individual spent in the refuge.

*Latency to Resume Feeding after Predator Cue*. In this setup, bats are given access to a feeding option. A predator cue (e.g., a vocalisation from a predatory bat species) is played back, and the behavioural response is quantified. “Time Until Feeding” measures the time from the predator cue playback until the bat resumes feeding. “Number of Feedings” records the total number of feeding events during the test. “Number Of Feeding Bouts” where a bout is defined as a cluster of feeding events separated by pauses shorter than 10 seconds. “Total Time Spent Feeding” refers to the sum of all time spent at the feeder (not just number of events). “Number of anti-predator behaviours” counts the sum of all presented anti predator behaviours, including, freezing events, defecation and aggression calls.

*Predator Cue*. This assay serves as a control test to identify whether or not the presented predator cue serves as a risk stimulus in the bats: the bats are exposed to a conspecific cue (conspecific call) and a predator cue, (vocalisation of a predatory bat species) and the anti-predator responses after both playbacks are recorded: “Number of Freezing events” (defined as at least two seconds of complete motionlessness), “Total Time Frozen”, “Number of Defecation” and “Aggression Calls”.

*General Activity in a Known Environment*. Here, bats are released into a flight cage that is familiar to them. We record “Number of Perches” which counts the number of times a bat lands or perches on the walls of the flight cage. “Total Time Spent Flying” quantifies the cumulative time the bat spends flying within the cage during the test. “Number of flights” refers to the number of discrete flight bouts from start to landing. “Number of section crossings” counts how many times the bats cross hypothetical sectors of the flight cage.

For every bat and every test, several additional covariates are recorded. Days in Captivity denotes how long the individual has already been in captivity at the time of the test. Capture Site specifies the location at which the bat was originally captured. The weight of each bat is also measured on the corresponding test day.

The fifth test, *Emergence from Roost in the Wild*, is conducted under natural conditions in an accessible roost box. After the standardized tests were conducted, the bats were equipped with Passive-Integrated-Transponders (PIT) – Tags, and a PIT-Tag-Reader was installed at the roost. In combination with a light barrier and a camera trap, this setup allows accurate determination of which individual bat leaves the roost at which time. The main variable recorded in this test is the time of emergence expressed as minutes after sunset, i.e. the number of minutes after local sunset at which a given bat leaves the roost. In our dummy dataset many of these values will be zero, representing cases where bats do not leave the roost during the sampling period, for example due to unfavourable weather conditions. We record individual emergence times from the roost during three separate nights, resulting in three repeated values for each individual.

**S2. Statistical protocol and dummy data**

Please visit our Open Science Framework project where you can find the full R project including scripts and dummy data: <https://osf.io/thdq7/overview?view_only=bfd6f9560d6f4f98ac452e115c8b838f>

This protocol does not aim to provide a comprehensive model selection framework, as decision guides already exist in the literature (e.g., Bolker et al. 2009; Nakagawa & Schielzeth 2010). Instead, we focus on the specific analytical steps and diagnostic checks most relevant to personality research and strongly encourage readers to consult these core references when selecting an appropriate model family for their data.

**S3. Results from dummy data**


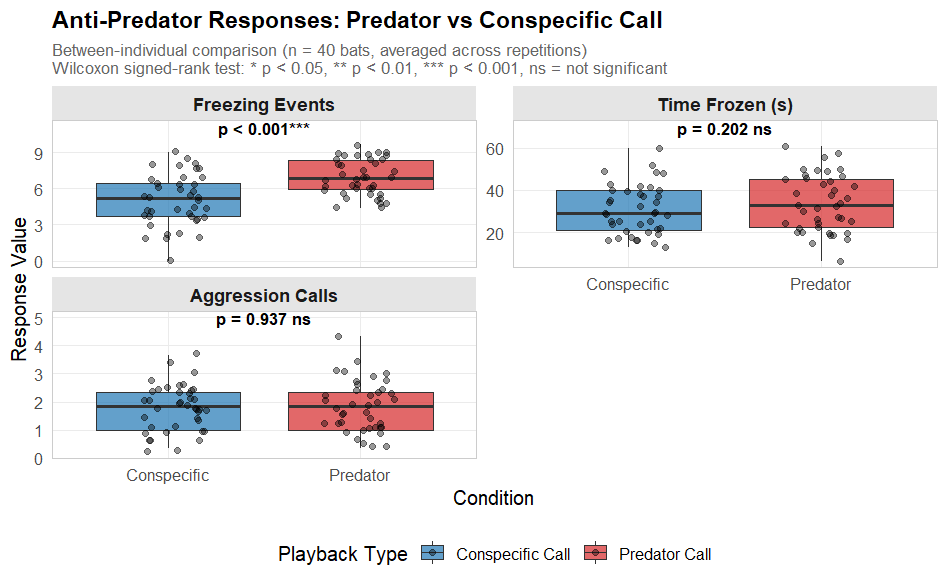


Fig S1: Step 3 – Wilcoxon signed rank test from predator cue test. Plots depicting the mean number of (1) freezing events, (2) total time frozen, and (3) number of aggression calls during the conspecific or predator condition, respectively (n=40). The grey horizontal bar in the boxplots depict the mean and the grey vertical bars outside the boxplots depict the 95% confidence intervals. P-values of the Wilcoxon Signed Rank Test are given in each panel for the respective test.

Table S1: Results from PCA for the three experimental assays. PCA was varimax rotated

| *Test* | *Variable* | *PC1* | *PC2* | *Communalities* | *Complexity* |
| --- | --- | --- | --- | --- | --- |
| **Refuge** | Time until head out | **0.90** | 0.12 | 0.82 | 1.0 |
|  | Time until full body out | **0.91** | 0.00 | 0.83 | 1.0 |
|  | Total time in refuge | **0.90** | -0.10 | 0.82 | 1.0 |
|  | Number of retractions | -0.01 | **0.90** | 0.81 | 1.0 |
|  | Number of returnings | -0.04 | **0.90** | 0.81 | 1.0 |
|  | Proportional Variance | 0.49 | 0.33 |  |  |
| **Feeding** | Time until feeding | **0.95** | 0.03 | 0.90 | 1.0 |
|  | Number of anti-predator behaviours | **-0.95** | 0.01 | 0.90 | 1.0 |
|  | Number of feedings | 0.08 | **0.90** | 0.82 | 1.0 |
|  | Total time spent feeding | **0.06** | **0.90** | 0.81 | 1.0 |
|  | Proportional Variance | 0.45 | 0.41 |  |  |
| **Activtiy** | Number of perches | **0.91** | -0.07 | 0.82 | 1.0 |
|  | Number of flights | **0.87** | -0.18 | 0.83 | 1.0 |
|  | Total time spent flying | -0.06 | **-0.86** | 0.82 | 1.0 |
|  | Number of section crossings | -0.19 | **0.79** | 0.81 | 1.0 |
|  | Proportional Variance | 0.41 | 0.35 |  |  |

Fig S2: Correlation between PCs across tests. Refuge_PC1 is representative for a refuge boldness axis, as it is mainly constructed by variables related to the time leaving the safe refuge. Feeding_PC1 is representative for a feeding boldness axis, as the variables loading onto this component are related to antipredator behaviour and the latency to resume feeding after the simulated predator attack. Panel A shows that refuge boldness and feeding boldness correlate, indicating that we can confirm convergent validity. Panel B shows now correlation of Refuge_PC1 with Activity_PC1 which represents the general activity axis of an individual, as the component is constructed by variables related to general locomotion. The lack of correlation in Panel B thus confirms discriminant validity.


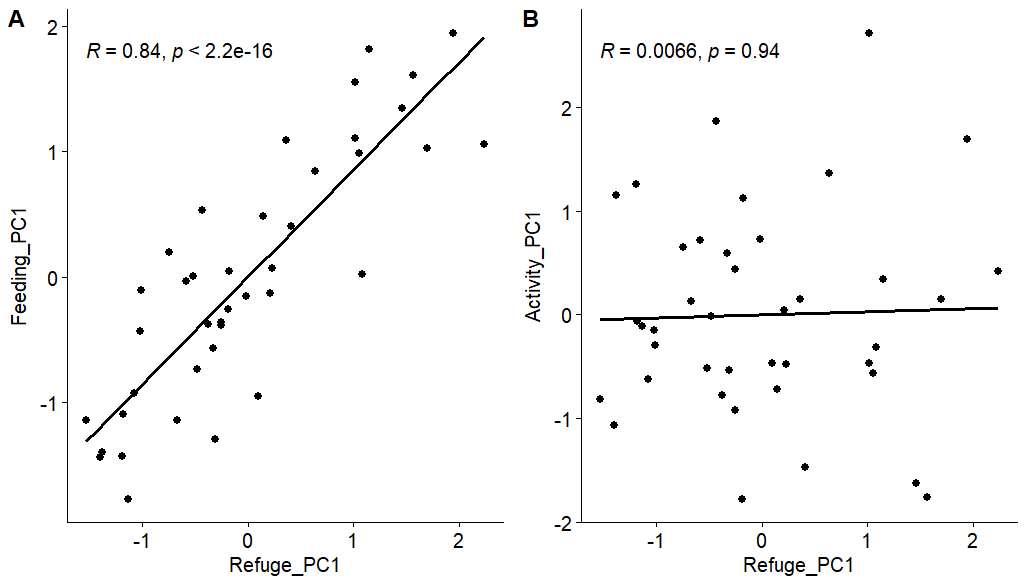


Table S2: Results from hurdle models used to assess the relationship between personality traits (captured as PCs) and latency to emerge from the natural roost. IRR = Incidence Rate Ratios, CI = Confidence Intervalls, p = p-value. Significant values are in bold. N=40, k=3

| **Latency to emerge [min]** | | | |  |
| --- | --- | --- | --- | --- |
| *Predictors* | *IRR* | *CI* | *p* |  |
| **Count Model** | | | |  |
| (Intercept) | 33.13 | 27.64 – 39.70 | **<0.001** |  |
| Refuge PC1 | 1.61 | 1.26 – 2.04 | **<0.001** |  |
| Refuge PC2 | 0.96 | 0.84 – 1.09 | 0.487 |  |
| Feeding PC1 | 1.40 | 1.09 – 1.80 | **0.008** |  |
| Feeding PC2 | 1.07 | 0.89 – 1.29 | 0.483 |  |
| (Intercept) | 6698.32 | 221.20 – 1748941.70 |  |  |
| **Zero-Inflated Model** | | | |  |
| (Intercept) | 1.31 | 0.88 – 1.95 | 0.190 |  |
| Refuge PC1 | 1.21 | 0.50 – 2.92 | 0.668 |  |
| Refuge PC2 | 0.67 | 0.43 – 1.05 | 0.082 |  |
| Feeding PC1 | 0.87 | 0.37 – 2.04 | 0.746 |  |
| Feeding PC2 | 2.72 | 1.70 – 4.37 | **<0.001** |  |
| Observations | 120 | | |  |
